# Acupuncture lowering blood pressure for secondary prevention of stroke: a study protocol for a multicenter randomized controlled trial

**DOI:** 10.1186/s13063-017-2171-5

**Published:** 2017-09-15

**Authors:** Yu-zheng Du, Xin-xin Gao, Cheng-Ting Wang, Hai-zhen Zheng, Yun Lei, Meng-han Wu, Xue-min Shi, Hai-peng Ban, Wen-long Gu, Xiang-gang Meng, Mao-ti Wei, Chun-xiao Hu

**Affiliations:** 1 Acupuncture and Moxibustion Department, First Teaching Hospital of Tianjin University of Traditional Chinese Medicine, No. 88, Changling Road, Xiqing District, Tianjin, 300000 China; 2 Emergency Department, First Teaching Hospital of Tianjin University of Traditional Chinese Medicine, Tianjin, China; 3 Tianjin University of Traditional Chinese Medicine, Tianjin, China; 4 Department of Epidemiology, Logistics College of Chinese People’s Armed Police Forces, Tianjin, China

**Keywords:** Acupuncture, Hypertension, Secondary prevention of stroke, RCT, Study protocol

## Abstract

**Background:**

Stroke is the prime cause of morbidity and mortality in the general population, and hypertension will increase the recurrence and mortality of stroke. We report a protocol of a pragmatic randomized controlled trial (RCT) using blood pressure (BP)-lowering acupuncture add-on treatment to treat patients with hypertension and stroke.

**Methods:**

This is a large-scale, multicenter, subject-, assessor- and analyst-blinded, pragmatic RCT. A total of 480 patients with hypertension and ischemic stroke will be randomly assigned to two groups: an experimental group and a control group. The experimental group will receive “*HuoXueSanFeng*” acupuncture combined with one antihypertensive medication in addition to routine ischemic stroke treatment. The control group will only receive one antihypertensive medication and basic treatments for ischemic stroke. *HuoXueSanFeng* acupuncture will be given for six sessions weekly for the first 6 weeks and three times weekly for the next 6 weeks. A 9-month follow-up will, thereafter, be conducted. Antihypertensive medication will be adjusted based on BP levels. The primary outcome will be the recurrence of stroke. The secondary outcomes including 24-h ambulatory BP, the TCM syndrome score, the Short Form 36-item Health Survey (SF-36), the National Institute of Health Stroke Scale (NIHSS), as well as the Barthel Index (BI) scale will be assessed at baseline, 6 weeks and 12 weeks post initiating treatments; cardiac ultrasound, carotid artery ultrasound, transcranial Doppler, and lower extremity ultrasound will be evaluated at baseline and 12 weeks after treatment. The safety of acupuncture will also be assessed.

**Discussion:**

We aim to determine the clinical effects of controlling BP for secondary prevention of stroke with acupuncture add-on treatment.

**Trial registration:**

ClinicalTrials.gov, ID: NCT02967484. Registered on 13 February 2017; last updated on 27 June 2017.

**Electronic supplementary material:**

The online version of this article (doi:10.1186/s13063-017-2171-5) contains supplementary material, which is available to authorized users.

## Background

Worldwide, stroke is a leading cause of death and disability [1–3]. A patient with a history of stroke is more vulnerable to recurrence [4, 5]^.^ Especially in China, the recurrence rate of stroke is higher than that of western countries [6, 7]. Recurrent stroke demonstrates higher mortality and disability rates than the previous stroke, which is a heavy burden on both health care resources and society in general [7, 8]. Ischemic stroke is the most common type of stroke and 72.7% of Chinese ischemic stroke patients also suffer from hypertension [6], an independent risk factor for stroke recurrence [9]. Therefore, it would be of great benefit in combating stroke recurrence to decrease blood pressure (BP).

Antihypertensive medications have been regularly used to reduce BP; however, low adherence to antihypertensive medications is prevalent due to various factors including prior experience of medication side effects [10], leading to poor BP control [11]. Uncontrolled BP maybe related to side effects or interactions of antihypertensives and this observation is more commonly noted in older populations [12, 13]. Moreover, a former meta-analysis has shown that only angiotensin-converting enzyme inhibition (ACEI) plus diuretics significantly decreases recurrent stroke events, and no significant association between BP reduction and all adverse outcomes was found [14]. It is apparent that the benefit of ACEI plus diuretics for secondary prevention of stroke is explained by mechanisms beyond the effects of lowering BP alone. The underlying mechanisms may be the divergent cardiovascular effects of ACEI [15], and the ability of diuretics to reduce proteinuria and protect the vascular endothelium [16]. Additionally, the presence of a J-shaped relationship between BP and risk of recurrent stroke is, to a certain degree, confirmed by previous research [17]. Under this context, in order to effectively regulate BP for reducing stroke recurrence, it is urgent to find an alternative that will affect multiple targets.

Acupuncture, with a long history of development in Chinese medicine, is becoming the focus of clinical researchers as an alternative for treating hypertension [18, 19]. Acupuncture-induced regulation of BP has been found to involve peripheral and central neural systems [20] and immune modulation mechanisms [21]. Its multiple-target features might explain the potential of acupuncture in effectively regulating BP to reduce stroke recurrence.

The synergistic BP-lowering effectiveness of acupuncture and antihypertensive agents has been proven, despite there formerly being insufficient evidence for acupuncture alone in lowering BP [22]. Recently, researchers have reported some positive results regarding sole acupuncture treatment for hypertension [23–25]. The reasons for this paradoxical situation could be disparities in acupuncture manipulation skills as well as other technical factors. Considering the limited reporting quality, the effect of pure acupuncture in lowering BP remains elusive.

Given that complementary and alternative interventions are recommended for mild and moderate hypertensive patients [26], we cautiously decided to select the hypertensive patients with uncontrolled BP (systolic BP ≥ 140 and < 160, or/and diastolic BP ≥ 90 and < 100 mmHg) who were taking one class of antihypertensive drugs to be the participants. The difference between the control and experimental groups is that in our experimental group, after a period of BP-lowering acupuncture add-on treatment, i.e., the “*HuoXueSanFeng*” needling method which has been proved to positively treat primary hypertension [27–33], if the BP falls below 140/90 mmHg, the prior antihypertensive drugs will be ceased, so as to observe the clinical effects of pure acupuncture on BP. Moreover, aiming to create a situation that is analogous to the real world, antihypertensive drugs will be adjusted based on the patients’ BP readings (detailed in the “Antihypertensive medication adjustment” section below) in both groups, respectively.

Altogether, our team conceived this high-quality, pragmatic randomized controlled clinical trial with the primary objective to determine whether BP-lowering acupuncture add-on therapy could decrease the recurrence of first-time ischemic stroke in patients during the 9-month follow-up period.

## Methods/design

### Study design and setting

**Fig. 1:**
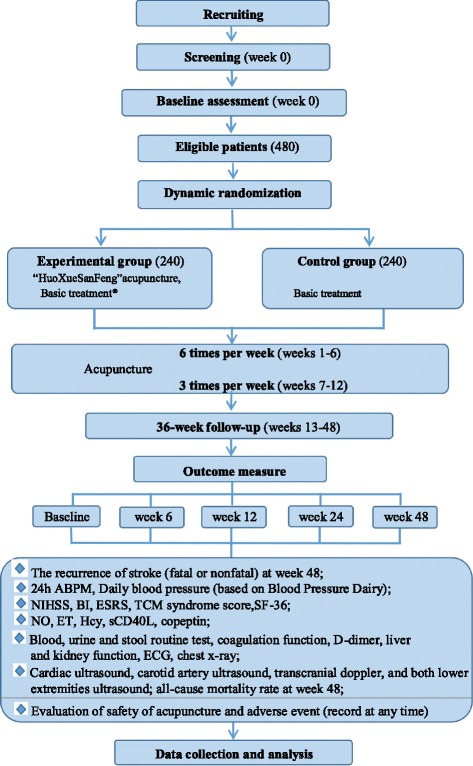
Study flowchart®: *XingNaoKaiQiao* acupuncture + other routine ischemic stroke interventions + one class of antihypertensive drug. *ABPM* ambulatory blood pressure monitoring, *NIHSS* National Institute of Health stroke scale, *BI* Barthel Index, *ESRS* Essen Stroke Risk Score, *TCM* traditional Chinese medicine, *SF-36* the medical outcome study (MOS) 36-item Short Form Health Survey, *NO* nitric oxide, *ET* endothelin, *Hcy* homocysteine, *sCD40L* soluble CD40 ligand, *ECG* electrocardiogram

Our study is a prospective, multicenter, subject-, assessor-, and analyst-blinded, pragmatic randomized controlled trial. The research period lasts from May 2015 to May 2020 (201507001-08). Five centers from five cities will be involved in recruitments, treatments, and assessments. Informed consent will be obtained after screening for eligibility, then baseline assessments will be conducted before randomization (details in Table 1). Eligible patients will be allocated, in a 1:1 ratio using a dynamic randomization procedure, into a BP-lowering acupuncture therapy group (experimental group) and a control group. After randomization, the patients in the experimental group will be additionally treated by the *HuoXueSanFeng* needling method (a BP-lowering regimen) plus the “*XingNaoKaiQiao*” needling method [34] (a formula for stroke) over 12 weeks, whereas the control group will be only given the *XingNaoKaiQiao* needling method for 12 weeks. Other routine ischemic stroke treatments in both groups will be provided. During the whole process of our study, the antihypertensive medications will be adjusted based on the BP level in the two groups (details displayed in the “Antihypertensive medication adjustment” section). The primary outcome will be all recurrent stroke cases over a follow-up period of 9 months after receiving 12-week acupuncture treatments. Our protocol presentation is in accordance with the Standard Protocol Items: Recommendations for Interventional Trials 2013 (SPIRIT 2013) guidelines. The study flowchart is presented as Fig. 1.

**Table 1:** Study schedule

Period	Screening	Baseline	Treatment (weeks 1–12)		Follow-up (weeks 13–48)	
Week	Week 0	Week 0	Week 6	Week 12	Week 24	Week 48
Eligibility	×					
Demographic medical history	×					
Informed consent		×				
Basic laboratory test®	×			×	×	
24-h ABPM		×	×	×		
TCM syndrome score, SF-36		×	×	×		
NIHSS, BI		×	×	×		
ESRS		×				×
Follow-up BP					×	×
Recurrence of stroke						×
NO, ET, Hcy, sCD40L, Copeptin		×		×	×	
ECG		×		×	×	
Chest X-ray		×				
Color Doppler ultrasound®		×		×		
All-cause mortality rate						×
Combined diseases and drugs	Record at any time					
Antihypertensive medication adjustment	Record at any time					
Adverse event	Record at any time					
Daily BP	Record everyday (based on BP diary)					
Safety of acupuncture	Record at any time					
Patients’ compliance				×		

Basic laboratory test®: blood, urine, and stool routine, coagulation function, D-dimer, liver and kidney function. Color Doppler ultrasound®: cardiac ultrasound, carotid artery ultrasound, transcranial Doppler of lower extremities ultrasound. *ABPM* ambulatory blood pressure monitoring, *SF-36* Short Form 36-item Health Survey, *NIHSS* National Institute of Health Stroke Scale, *BI* Barthel Index, *ESRS* Essen Stroke Risk Score, *BP* blood pressure, *NO* nitric oxide, *ET* endothelin, *sCD40L* soluble CD40 ligand, *ECG* electrocardiograph, *Hcy* homocysteine, *TCM* traditional Chinese medicine

### Participants

We will recruit 480 eligible patients from (1) The First Teaching Hospital of Tianjin University of traditional Chinese medicine (TCM), (2) Longhua Hospital Shanghai University of Traditional Chinese Medicine, (3) Chongqing Traditional Chinese Medicine Hospital, (4) Mianyang Hospital of Traditional Chinese Medicine, and (5) Shenzhen Bao’An Traditional Chinese Medicine Hospital Group. The eligible patients must satisfy the criteria defined below. Patients who express a general interest in taking part in the study will meet with one of the study personnel to be informed of all necessary information in oral and written form. After fulfilling the criteria and signing the consent, the study personnel will assign the patient to the acupuncturist and the neurologist who is in charge of the patient.

### Inclusion criteria

Patients will be further screened for eligibility if they fulfill all of the following inclusion criteria: (1) first-ever ischemic stroke that has been confirmed by cranial computed tomography (CT) or magnetic resonance imaging (MRI), with stroke duration over 2 weeks and less than 6 weeks; (2) BP below 160/100 mmHg on using one antihypertensive medication except the fixed-dose combinations, for at least 2 weeks; (3) aged 35 to 70 years; and (4) voluntary informed consent signed.

### Exclusion criteria

Patients will be excluded if they have any one of the following: (1) secondary hypertension, (2) antihypertensive medication general use for reasons other than BP control, such as long-term need for beta-blockers or diuretics or non-dihydropyridine calcium channel antagonists for cardiac conditions, (3) BP below 140/90 mmHg when taking one hypotensive drug, (4) other coexisting nervous system diseases like epilepsy and peripheral nerve injuries, (5) severe hematopoietic system conditions, coagulation dysfunction, and cancers, (6) diabetic nephropathy, severe liver and renal function impairment, severe heart or lung dysfunction, and arrhythmia, (7) local infection at or around the acupoint, (8) pregnant or lactating women, and (9) recruitment into other clinical trials within 1 month.

### Ethical issues

Our protocol complies with the principles of the Declaration of Helsinki and has been approved by the central Independent Ethics Committee (IEC) of The First Affiliated Hospital of Tianjin University of Traditional Chinese Medicine (TCM) for the various centers (reference number: TYLL2016[K]NO.007). We have also registered at ClinicalTrials.gov (NCT02967484). The overall supervision of our trial will be in the charge of the central IEC of the First Affiliated Hospital of Tianjin University of TCM; any change in the protocol will be submitted to, and decided by, the Ethics Committee.

### Recruitment strategies

Firstly, all the patients will be enrolled from the inpatient departments of the acupuncture and moxibustion sections of each branch center, to fulfill the completion of the consistent acupuncture treatments for 6 weeks. We will also use advertisements, such as printed recruitment posters, in each participating hospital, and the various social media platforms to distribute the trial information.

### Informed consent

The details of our study, including trial objectives, characteristics, probable benefits and risks, other available treatment alternatives, and the subjects’ rights as well as obligations as stated in the Declaration of Helsinki, will be made clear to the patients by a study personnel who has their grouping code, and their agreement will be required. After obtaining the written informed consent, the subjects will be enrolled in the study.

During the process of the trial, if new points regarding the study ethics emerge, written revisions regarding the informed consent will be handled by the Ethics Committee and, after their approval, the individuals’ consent will be requested again. In case of the patients’ withdrawal, their available data will be kept for the final analyses.

### Randomization and allocation concealment

All the eligible participants will be randomized into the experimental group or the control group, in a 1:1 ratio, using minimization, a dynamic random allocation technique by the MINIM program installed in each separate center. The baseline prognostic factors related to stroke recurrence, including age, categorized as 35–50, 51–65, and 66–70 years), gender, smoking status, diabetic status and atrial fibrillation, classified as “no” or “yes with/without anti-coagulation,” will be balanced between groups according to the minimization procedures. The computer-generated randomization grouping code will be offered after the recruitment of the eligible patients so that allocation concealment should be ensured.

### Interventions (Table 2)

**Table 2:** Protocol form

Item	Control group	Experimental group
Aim	To testify the efficacy of the acupuncture method of *HuoXueSanFeng* on stroke recurrence among ischemic stroke patients with hypertension	
Interventions	1 type of antihypertensive drug (may be adjusted based on the BP levels) + *XingNaoKaiQiao* acupuncture method + other routine care for stroke	*HuoXueSanFeng* acupuncture method + *XingNaoKaiQiao* acupuncture method + 1 type of antihypertensive drug (may be adjusted based on the BP levels) + other routine care for stroke
Intervention time	Continuous treatment period: 6 times a week, 6 weeks Consolidation treatment period: three times a week, 6 weeks	
Observation time	Before treatment, 6 weeks, 12 weeks, 24 weeks, and 48 weeks after treatment	
Researchers	To ensure the optimal effects of acupuncture stimulation, our therapists will be required to have clinical experience of acupuncture treatments for at least 8 years	
Materials	Disposable, sterile needles, 0.25 mm in diameter and 40 mm in length (HWATO, Suzhou, China)	

*BP* blood pressure

Both groups receiving acupuncture treatments ensures the blinding of subjects.

### Experimental group

Patients in the experimental group will be given HuoXueSanFeng acupuncture therapy, in addition to the control group interventions. To ensure the optimal effects of acupuncture stimulation, all the therapists are required to have clinical experience of acupuncture treatments for at least 8 years.

Disposable, sterile needles with a diameter of 0.25 mm and a body length of 40 mm (HUATO, Suzhou, China) will be used. Patients are asked to adopt the supine position. Acupoints used here are bilateral *Renying* (ST9), *Hegu* (LI4), *Quchi* (LI11), *Taichong* (LR3), and *Zusanli* (ST36). The acupoint locations with reference to the World Health Organization (WHO) standards in the Western Pacific Region, and the corresponding manipulations of the aforementioned acupoints are listed in Table 3, which accord with those described in a previous paper [32]. All the manipulations will be applied for 1 min and the needles are retained for 30 min.

**Table 3:** Acupoint form

Treatment methods	Points	Location	Acupuncture method
Acupuncture method of *HuoXueSanFeng*	*Renying* (ST9)	In the neck next to the thyroid cartilage, the anterior border of sternocleidomastoid, at the common carotid artery pulsation site	Using the twisting technique of the reinforcing method of the second definition from Xue-min Shi academician, namely low amplitude (torsional amplitude less than 90°), high-frequency (120–160 times/min), the operation lasting for 1 min
	*Hegu* (LI4)	In the back of the hand, between the first and second metacarpals, at the midpoint of the second metacarpal radially	Using the twisting technique of the first definition of the reducing method from Xue-min Shi, academician
	*Taichong* (LR3)	In the dorsum of the foot, at the head of the first metatarsal space	Using the twisting technique of the first definition of the reducing method from Xue-min Shi, academician
	*Quchi* (LI11)	In the lateral end of the cubital crease, at the midpoint of the ligature between the end point of the cubital crease and the lateral epicondyle of the humerus with the elbow bent	Using the twisting technique of the first definition of the reinforcing method from Xue-min Shi, academician
	*Zusanli* (ST36)	In the anterior lateral of crus, 3 B-cun under *Dubi* (ST35), one transverse finger width from the anterior tibial border	Using the twisting technique of the first definition of the reinforcing method from Xue-min Shi, academician
Acupuncture method of *XingNaoKaiQiao*	*Neiguan* (PC6)	In the volar aspect of the forearm, at the junction of *Quze* (PC 3) and *Daling* (PC7), 2 B-cun above the wrist crease in the space between tendon palmaris longus and vagina tendinis musculi flexoris carpi radialis	Using the combination of the reducing method of twirling lifting and thrusting needles, the operation lasts for 1–3 mins
	*Shuigou* (DU26)	In the face, at the intersection point of the upper 1/3 and middle 1/3 of the philtrum groove	Using the reducing method of “sparrow-pecking” until tears fall or appear in the eyes
	*Sanyinjiao* (SP6)	In crus inside, 3 B-cun above the upper border of the medial malleolus of the ankle at the posterior border of the tibia	Using the reinforcing method of twirling, lifting, and thrusting needles, until the lower limb consecutively twitches 3 times
	*Jiquan* (HT1)	At the apex of the axilla, the site of the pulse of the axillary artery	Using the reducing method of twirling, lifting, and thrusting needles, until the upper extremity twitches 3 times
	*Chize* (LU5)	In the cubital crease, at the depression of the bicipital muscle tendon radialis at the elbow	Using the reducing method of twirling, lifting, and thrusting needles, until the needling feeling is felt from the elbow joint to the fingers; or manual extorsion 3 times
	*Weizhong* (BL40)	At the midpoint of the popliteal crease, at the midpoint between the femoral biceps tendon and the semitendinosus tendon	Using the reducing method of twirling, lifting, and thrusting needles, until the lower extremity twitches 3 times

B-cun: proportional bone cun. This method divides the height of the human body into 75 equal units. Using joints on the surface of the body as the primary landmarks, the length and width of every bodily region is measured by such proportions

The patients will be provided with *HuoXueSanFeng* acupuncture treatments six times per week in the first 6 weeks, referred to as the consistent treatment period. The consolidation period consists of three sessions of *HuoXueSanFeng* acupuncture treatments weekly in the following 6 weeks with a total of 12 weeks of *HuoXueSanFeng* acupuncture treatments performed in the experimental group.

### Control group

The control group will receive one antihypertensive medication on the basis of ischemic stroke routine care. In our trial, basic ischemic stroke medical care includes 12 weeks of *XingNaoKaiQiao* needling therapy plus other forms of routine stroke care. Twelve weeks of *XingNaoKaiQiao* needling therapy should also aid in the improvement of compliance in both groups for its high acceptance and remarkable effectiveness for stroke. All medications must be recorded in the drug combination form, including the types, dosage, usage frequency and time, etc., in order to provide data for analysis and the report summary. The acupuncture needle is the same with that used in *HuoXueSanFeng* acupuncture treatments. The acupoint locations and manipulations are presented in Table 3. No common acupoints are used in the two formulas. The retention time and frequency of treatments are identical to *HuoXueSanFen*g acupuncture therapy. During the entire process of our study, the control-group patients are prohibited from receiving any BP-lowering acupuncture therapy, and any violations will be recorded.

### Antihypertensive medication adjustment

After randomization, an electronic BP machine and a BP diary notebook will be delivered to all patients for measuring and recording their BP levels. During the first 6-week inpatient treatments, they will be instructed how to use the machine accurately. BP will be measured within 1 h after morning waking before taking antihypertensive medications, at 3 h after taking antihypertensive medications, at 4 p.m. and before bedtime over the entire process. On each occasion, three repeated measurements of supine BP, following a rest of at least 15 min, will be conducted at 1-min intervals. The antihypertensive medication classes in both groups will be recorded. Lifestyle advice regarding hypertension and stroke will be given to the patients. In the experimental group, if the patients have a mean BP level below 140/90 mmHg for three consistent days, they will receive solely acupuncture treatment; thereafter, once their BP levels increase to ≥ 140 mmHg systolic and/or ≥ 90 mmHg diastolic for 1 month, prior antihypertensive medications should be re-dispensed. Otherwise, if BP levels are ≥ 160 mmHg systolic and/or ≥ 100 mmHg diastolic for three consistent days after initiating *HuoXueSanFeng* acupuncture treatments or over the follow-up period, additional antihypertensives should be prescribed. All the medication adjustments will be filled out in the Case Report Forms (CRFs).

For the control group, when the average systolic BP increases to ≥ 160 mmHg and/or diastolic BP ≥ 100 mmHg lasting for three continuous days in the study period, other antihypertensive drugs will be prescribed in the opinion of the treating physicians, and these will be documented for final analyses.

### Outcome assessments

The assessors are blinded to grouping. The primary outcome will be all cases of recurrent stroke over a follow-up period of 9 months after receiving 12-week acupuncture treatments. Secondary endpoints, such as 24-h ambulatory BP, TCM syndrome score, Short Form 36-item Health Survey (SF-36) results, National Institute of Health Stroke Scale (NIHSS) and Barthel Index (BI) scale scores will be assessed at baseline, 6 weeks, and 12 weeks post initiating treatments; serum nitric oxide (NO), endothelin (ET), homocysteine (Hcy), soluble CD40 ligand (sCD40L), and copeptin levels and cardiac ultrasound, carotid artery ultrasound, transcranial Doppler, and lower extremity ultrasound results will be evaluated at baseline and 12 weeks after treatment. Other safety indicators including baseline general physical examination, blood, urine, and stool routine tests, coagulation function, D-dimer level, liver and kidney function, electrocardiograph as well as a chest X-ray will also be examined 12 weeks after randomization. At 6 months after randomization, blood, urine, and stool routine tests, coagulation function, D-dimer level, liver and kidney function, electrocardiograph, NO, ET, Hcy, sCD40L, and copeptin tests will be re-checked, with some hope of improving patients’ adherence to treatment. All-cause mortality will also be evaluated during the follow-up period. The Essen Stroke Risk Score (ESRS) will be additionally assessed at baseline and 1 year post randomization.

### Follow-up

After finishing the acupuncture treatments, patients will be followed up monthly via telephone for 1 year post randomization. Stroke recurrence, all-cause deaths and BP control will be investigated and recorded in the CRFs. The patients will be required to use the BP diaries to record their BP four times daily. If the BP is ≤ 160 mmHg systolic and/or ≤ 100 mmHg diastolic for 1 month, the patient will be advised to take antihypertensive medications, which will be documented in the CRF.

### Sample size and statistical methods

The sample size calculation of the study was based on China’s Ministry of Health data suggesting that at least 197 patients are required in each group for the final analysis to achieve a power of 80% to demonstrate a recurrence rate of 7%, rather than 16% resulting from *HuoXueSanFeng* acupuncture (data not shown, please see Additional file 1), at a two-sided significance level of 5%. Given a projected dropout rate of 15%, we aimed to recruit 480 patients for the study.

Statistical analysis will be conducted by a third party who is unaware of the treatment allocation. Data will be analyzed with SAS 9.1 and SPSS 19.0 software packages. Two-sided *P* < 0.05 represents statistical significance for all the analyses. Both intention-to-treat analysis (ITT) and per-protocol (PP) analysis will be performed. We will compare the result of ITT analysis with that of PP analysis to determine whether the results are consistent. For the ITT analysis, missing data will be handled by Last Observation Carry Forward rules, in brief, the finally observed data will be used for replacement of the missing primary variables.

Baseline demographic characteristics, including individual variables, such as age, sex, history of atrial fibrillation with or without current anti-coagulation medication, history of diabetes and smoking, and other baseline values, will be expressed with descriptive statistics for the two groups. For the primary outcome, recurrent stroke events, and secondary outcomes, the mortality rate and detailed causes (stroke or non-stroke), will be described as percentage (%), between-group difference will be analyzed using the chi-square test or Fisher’s exact test. For filling in the daily BP diary, it is required to measure BP four times each day at baseline before treatment, 2 weeks, 6 weeks, 12 weeks, 6 months, and 1 year after treatment. We will compare between-group or within-group values at the indicated time points. For highly effective use of the daily BP data, on one hand, we will collect all the values of the measurements at the same time in the day until each predesigned time point for comparison and, on the other hand, we will collate the four daily measurements to yield an average value in a day for later comparison at the time points mentioned above. Other secondary endpoints at different time points will also be compared. All the above different time-point assessments will be analyzed by repeated measures analysis of variance (ANOVA). Adverse events in each group will be documented as percentage (%) for safety assessments using the chi-square test or Fisher’s exact test.

### Patient safety

In the process of acupuncture treatment, possible adverse events (AEs) including subcutaneous hematoma, infection, alcohol allergy, etc., will be observed in detail and documented in the CRFs. Causality between acupuncture and AEs will be assessed. If serious AEs do occur, the grouping will be un-blinded and we will report the AE to the project leader (PL) and Ethics Committee immediately, who will make a decision on whether the participant needs to be withdrawn from the study, or whether the trial should be adjusted or terminated. For those who suffer harm from trial participation, we will treat them free of charge until they recover.

### Data management and quality control

The teaching and research office of epidemiology and statistics of the Chinese People’s Armed Police Force Logistics College is fully responsible for the data management of the project. Prior to the project, the center has been involved in the design of the project, which has been discussed twice monthly with the principle experts in the State Administration of Traditional Chinese Medicine of the People’s Republic of China, and project implementation throughout the process has successfully completed quality control and data management scrutiny. A specific research assistant (RA), who is masked to treatment allocation, will enter the clinical data. Data will be obtained by direct questions to patients, from medical records, and a the patient’s relative or proxy. The personal information of the patients will be kept in the specific files with pre-set codes. Entering data into CRFs and other essential skills was covered during the training session prior to study commencement.

All the researchers will accept centralized and unified training before research implementation to help them become familiar with, and master, the diagnosis, treatment measures and basic assessment skills, in order to improve the researchers’ internal observation consistency. A three-level inspector appointed for the national Chinese Medicine Clinical Research Base Unit, First Teaching Hospital of Tianjin University of TCM, and each subcenter will regularly carry out quality monitoring and supervision throughout the study.

### Dissemination

We plan to publish several Chinese and English articles, and hold one or two seminars yearly to disseminate part of our new findings to health care professionals, the public, and other relevant groups. On the other hand, the participants will be instructed on how to better manage their condition after the results yield useful information. After our trial finishes, we will make an effort to share our findings with the experts from other Chinese medical fields, hoping to benefit them by the drafting of stroke and hypertension guidelines incorporated into the features of TCM.

### Biological specimens

The patients’ blood samples for the inpatient routine tests will be collected and analyzed as usual. For testing serum NO and ET concentrations, about 3–5 ml venous blood will be collected; after centrifugation, the remaining serum will be subpacked into frozen tubes, followed by concise labeling, then kept for no more than 6 months in a refrigerator at −80 °C until final analysis by the chemical reagent corporation.

## Discussion

Acupuncture within Chinese medicine has become a visible international brand of China. Both stroke and hypertension are heavy global disease burdens and both have alarmingly high comorbidities [35] which are associated with a poorer prognosis. Antihypertensive medications have been commonly used for preventing stroke recurrence. However, evidence-based medicine data regarding secondary prevention of stroke by antihypertensives is controversial [14, 36]. Therefore, it is of great clinical significance that our study aims to decrease recurrent stroke using acupuncture, in the hope of increasing western knowledge of acupuncture.

As early as in 1983, the WHO recommended non-drug therapy as the preliminary and auxiliary treatment of mild-to-moderate hypertension [26]. With the enhancement of people’s drug safety awareness, the proportion of non-drug therapy in the treatment of hypertension has gradually increased. Acupuncture, with its advantages of convenience, satisfactory effects and few adverse reactions [37], is attracting increasing clinical attention and is also becoming one of the hotly debated topics in the study of alternative medicine worldwide.

The acupuncture method of *XingNaoKaiQiao* for the treatment of stroke was launched in 1972 by the first, and to date, only Chinese academician of engineering in the acupuncture discipline, Xue-min Shi, who devoted himself to introducing acupuncture to the whole world and developed an international academic team that cooperates with Germany, France, Japan, and Singapore, among other countries. For its internationally substantial contributions, *XingNaoKaiQiao* needling therapy for stroke has been recognized as another special acupuncture technique in addition to acupuncture anesthesia by foreign health care researchers. Besides the confirmed effects for stroke by our own team, the others also reported its encouraging results for stroke patients [38]. With the great success of *XingNaoKaiQiao*, academician Xue-min Shi gained deeper insight into secondary stroke prevention, and established another acupuncture treatment for hypertension called *HuoXueSanFeng*, the BP-lowering acupuncture regimen used in our trial. Our previous researches have shown that this acupuncture method reduces BP for primary hypertension patients, and its immediate and long-term antihypertensive efficacy is significant for either BP-lowering or improving the patients’ clinical symptoms [27–33]. So, here we also want to investigate the BP-controlling effect of *HuoXueSanFeng*, especially regarding its sole efficacy, in a larger sample size population. In this study, both *XingNaoKaiQiao* and *HuoXueSanFeng* needling therapy are performed over the 12-week treatment period; *XingNaoKaiQiao* therapy is a general treatment for stroke with a high credibility, its free treatment here will enhance adherence with participation. Additionally, because both groups are needled, subject blinding should be ensured, minimizing the placebo effect of acupuncture.

Of note, in our trial, we cautiously selected three secondary endpoints, namely, Hcy, sCD40L, and copeptin levels, which were closely related to stroke recurrence. Elevated Hcy levels have been reported to be indicative of an increased risk for ischemic strokes and recurrent strokes [39]. sCD40L appears to be an independent predictor for stroke recurrence [40]. Copeptin has been studied to be a predictor of both severity at admission and 1-year stroke recurrence in stroke patients [41]. Assessing the influence of our study treatment on them might be reflective of the effects on stroke recurrence via acupuncture lowering hypertension.

The multicenter approach of our study allows more generalisable interpretation of the results and the usage of standardized, cross-culturally adapted assessment allows comparison and pooling of all study data from different trial centers. Considering methodological and ethical reasons, acupuncture will be delivered to both groups to prevent bias through potential placebo effects. To improve operation and observation consistency, the research sets strict rules that must be followed.

The unavoidable weaknesses of our trial are as follows: (1) the five hospitals are located in different cities, so the standard of acupuncture manipulation will differ between them. Therefore, strict training and supervision will be implemented to ensure unified and consistent operation as far as possible, (2) despite the subject- and assessor-blinding, there are still some patients who will likely know which group they are in; therefore, we would keep the patients separate when receiving treatment to avoid them talking to each other; (3) after completion of the 12-week treatment, a 9-month follow-up will be conducted. This time span means that participant compliance could be a problem. Accordingly, we have designed the BP diary and regular telephone follow-up to try to minimize this.

## Additional files

Additional file 1: Sample size calculation process. (DOC 31 kb)

Additional file 2: SPIRIT 2013 Checklist. (DOC 139 kb)

## Abbreviations

ABPM: Ambulatory blood pressure monitoring
ACEI: Angiotensin-converting enzyme inhibition
AEs: Adverse events
ANOVA: Analysis of variance
BI: Barthel Index
BP: Blood pressure
CRFs: Case Report Forms
ECG: Electrocardiograph
ESRS: Essen Stroke Risk Score
ET: Endothelin
Hcy: Homocysteine
IEC: Independent Ethics Committee
ITT: Intention-to-treat
NIHSS: National Institute of Health Stroke Scale
NO: Nitric oxide
PL: Project leader
PP: Per-protocol
RA: Research assistant
RCT: Randomized controlled trial
sCD40L: Soluble CD40 ligand
SF-36: Short Form 36-item Health Survey
SPIRIT 2013: Standard Protocol Items: Recommendations for Interventional Trials 2013
TCM: Traditional Chinese medicine.

## References

[1] Party ISW (2004). National Clinical Guidelines for Stroke.

[2] Chen Z (2008). The third national survey on the cause of death.

[3] Liu M, Wu B, Wang WZ, Lee LM, Zhang SH, Kong LZ (2007). Stroke in China: epidemiology, prevention, and management strategies. Lancet Neurol..

[4] Roger VL, Go AS, Lloyd-Jones DM, Benjamin EJ, Berry JD, Borden WB (2012). Heart disease and stroke statistics—2012 update: a report from the American Heart Association. Circulation..

[5] Kernan WN, Ovbiagele B, Black HR, Bravata DM, Chimowitz MI, Ezekowitz MD (2011). Guidelines for the prevention of stroke in patients with stroke or transient ischemic attack: a guideline for healthcare professionals from the American Heart Association/American Stroke Association. Stroke..

[6] Wang Y, Xu J, Zhao X, Wang D, Wang C, Liu L (2013). Association of hypertension with stroke recurrence depends on ischemic stroke subtype. Stroke..

[7] Hardie K, Hankey GJ, Jamrozik K, Broadhurst RJ, Anderson C, Hardie K (2004). Ten-year risk of first recurrent stroke and disability after first ever stroke in the Perth Community stroke study. Stroke..

[8] Mohan KM, Crichton SL, Grieve AP, Rudd AG, Wolfe CD, Heuschmann PU (2009). Frequency and predictors for the risk of stroke recurrence up to 10 years after stroke: the South London Stroke Register. J Neurol Neurosurg Psychiatry..

[9] Wu L, Wang A, Wang X, Zhao X, Wang C, Liu L (2015). China National Stroke Registry investigators. Factors for short-term outcomes in patients with a minor stroke: results from China National Stroke Registry. BMC Neurol.

[10] Lulebo AM, Mutombo PB, Mapatano MA, Mafuta EM, Kayembe PK, Ntumba LT (2015). Predictors of non-adherence to antihypertensive medication in Kinshasa, Democratic Republic of Congo: across-sectional study. BMC Res Notes.

[11] Matsumura K, Arima H, Tominaga M, Ohtsubo T, Sasaguri T, Fujii K (2013). Impact of antihypertensive medication adherence on BP control in hypertension: the COMFORT study. QJM..

[12] Brescacin L (2011). Is it possible to apply secondary stroke prevention guidelines to very old populations?. Cardiovasc Hematol Disord Drug Targets..

[13] Sanossian N, Ovbiagele B (2009). Prevention and management of stroke in very elderly patients. Lancet Neurol..

[14] Wang WT, You LK, Chiang CE, Sung SH, Chuang SY, Cheng HM (2016). Comparative effectiveness of BP-lowering drugs in patients who have already suffered from stroke: traditional and Bayesian network meta-analysis of randomized trials. Medicine (Baltimore).

[15] Strauss MH, Hall AS (2016). The divergent cardiovascular effects of angiotensin converting enzyme inhibitors and angiotensin receptor blockers on myocardial infarction and death. Prog Cardiovasc Dis..

[16] Roush GC, Sica DA (2016). Diuretics for hypertension: a review and update. Am J Hypertens..

[17] Odden MC, McClure LA, Sawaya BP, White CL, Peralta CA, Field TS (2016). Achieved BP and outcomes in the secondary prevention of small subcortical strokes Trial. Hypertension..

[18] Li J, Zheng H, Zhao L, Li Y, Zhang Y, Chang XR (2013). Acupuncture for patients with mild hypertension: study protocol of an open-label multicenter randomized controlled trial. Trials..

[19] Kim JH, Jung HJ, Kim TH, Lee S, Kim JE, Kang KW (2013). Auricular acupuncture for prehypertension and stage 1 hypertension: study protocol for a pilot multicentre randomized controlled trial. Trials..

[20] Longhurst JC, Tjen-A-Looi S (2013). Acupuncture regulation of BP: two decades of research. Int Rev Neurobiol..

[21] Yu Z, Wu QF, Liang FR (2014). Considerations about mechanisms of acupuncture therapy for improving hypertension by regulating immune system. Zhen Ci Yan Jiu..

[22] Zhao XF, Hu HT, Li JS, Shang HC, Zheng HZ, Niu JF (2015). Is acupuncture effective for hypertension? A systematic review and meta-analysis. PLoS One.

[23] Liu Y, Park JE, Shin KM, Lee M, Jung HJ, Kim AR (2015). Acupuncture lowers BP in mild hypertension patients: a randomized, controlled, assessor-blinded pilot trial. Complement Ther Med..

[24] Li P, Tjen-A-Looi SC, Cheng L, Liu D, Painovich J, Vinjamury S (2015). Long-Lasting Reduction of BP by electroacupuncture in patients with hypertension: randomized controlled trial. Med Acupunct..

[25] Yang X, Liu W (2015). Primary hypertension treated with acupuncture combined with auricular point sticking: a randomized controlled trial. Zhongguo Zhen Jiu..

[26] Wallace JP (2003). Exercise in hypertension. A clinical review. Sports Med.

[27] Gao XX, Ma F, Zhao Q, Zhang Y, Du YZ (2016). The efficacy of “*HuoXueSanFeng*” acupuncture to reduce BP of stroke patients with hypertension. Zhongguo Zhenjiu..

[28] Wang ZR, Rong WZ, Jin SS, Gu WL, Ma F, Li XZ (2016). Regulation of activating blood and dispersing wind, harmonizing liver and spleen acupuncture method on morning blood pressure and blood pressure variability in patients with hypertension complicated with cerebral infarction. Liaoning Journal of Traditional Chinese Medicine..

[29] Gu WL, Liu CX, Wang ZR, Gong FM, Wang T, Du YZ (2015). Effects of blood activating wind dissipating acupuncture on blood pressure of prehypertension patients. Zhongguo Zhong Xi Yi Jie He Za Zhi..

[30] Yin C, Du YZ (2012). Observation of anti-hypertensive effect on primary hypertension treated with acupuncture at *Ren-ying* (ST9) mainly. Zhongguo Zhen Jiu..

[31] Shen PF, Shi XM (2010). Evaluation of antihypertensive effect on essential hypertension from acupuncture by ambulatory blood pressure monitoring. Mode Tradit Chin Med Mater Med..

[32] Zhang L, Shen P, Wang S (2014). Acupuncture treatment for hypertension: a case study. Acupunct Med..

[33] Zheng H, Zhao X, Du Y, Shi X (2016). Acupuncture for blood pressure control in stroke patients: case reports. Forsch Komplementmed..

[34] Shi XM, Shi XM (1998). “Xing Nao Kai Qiao” needling method formula. Stroke and “Xing Nao Kai Qiao” needling method.

[35] Lawes CM, Vander Hoorn S, Rodgers A (2008). Global burden of blood-pressure-related disease, 2001. Lancet..

[36] Katsanos AH, Filippatou A, Manios E, Deftereos S, Parissis J, Frogoudaki A (2017). Blood pressure reduction and secondary stroke prevention: a systematic review and metaregression analysis of randomized clinical trials. Hypertension..

[37] Park SU, Ko CN, Bae HS, Jung WS, Moon SK, Cho KH (2009). Short-term reactions to acupuncture treatment and adverse events following acupuncture: a cross-sectional survey of patient reports in Korea. J Altern Complement Med..

[38] Zhang S, Wu B, Liu M, Li N, Zeng X, Liu H (2015). Acupuncture efficacy on ischemic stroke recovery: multicenter randomized controlled trial in China. Stroke..

[39] He Y, Li Y, Chen Y, Feng L, Nie Z (2014). Homocysteine level and risk of different stroke types: a meta-analysis of prospective observational studies. Nutr Metab Cardiovasc Dis..

[40] Li J, Wang Y, Lin J, Wang D, Wang A, Zhao X (2015). Soluble CD40L is a useful marker to predict future strokes in patients with minor stroke and transient ischemic attack. Stroke..

[41] Tang WZ, Wang XB, Li HT, Dong M, Ji X. Serum copeptin predicts severity and recurrent stroke in ischemic stroke patients. Neurotox Res. 2017. doi:10.1007/s12640-017-9754-5. 10.1007/s12640-017-9754-5 28555260

