# Supplementary material for: Acupuncture lowering blood pressure for secondary prevention of stroke: a study protocol for a multicenter randomized controlled trial
Source: Trials. 2017 Sep 15;18:428. doi: 10.1186/s13063-017-2171-5 (PMC5603044; doi:10.1186/s13063-017-2171-5)
Supplement: Supplementary file 1 — Sample size calculation process. (DOC 31 kb) [file 13063_2017_2171_MOESM1_ESM.doc]

The clinical trial we carried out, named *"HuoXueSanFeng" acupuncture intervention on blood pressure regulation in cerebral infarction patients* (project number: JDZX2012151) lasted from January 2013 to December 2014. It was approved by the State Administration of traditional Chinese medicine as special scientific research project for base construction. The trial adopted randomised controlled design, took in hypertension patients complicated with cerebral infarction as subjects, and introduced "HuoXueSanFeng" acupuncture method on hypertension regulation. All of the 72 subjects were from the First Teaching Hospital of Tianjin University of Traditional Chinese Medicine, randomly divided into experimental group and control group. The control group, on the basis of “XingNaoKaiQiao” acupuncture treatment for stroke, took oral antihypertensive medicine; the experimntal group, except for the same intervention of control group, received "HuoXueSanFeng" acupuncture antihypertensive therapy for 30 minutes each time, once a day, 6 times a week (a total of 30 times) and was followed-up one year later. The regulation effect on cerebral infarction patients’ blood pressure was evaluated comprehensively by 24h ambulatory blood pressure monitoring as well as the points of traditional Chinese medicine main syndrome, quality of life scale and stroke recurrence rate. Among 72 cases, 11 cases dropped out because of poor compliance, leaving 31 cases in experimental group and 30 cases in control group. 61 patients were followed up after 1 year, with 2 cases of recurrence observed, meaning the recurrence rate was 6.5%. Based on this data, we speculate the recurrence rate of the whole was 7%. According to the data of Ministry of Health, the one-year ischemic stroke recurrence rate in China was 16%. The formula: was used for sample size calculation(P_0_=16%，P_1_=7%，P=11.5%). After two-sided test, Z_α_=0.05=1.96，Z_β_=0.2=1.28 (power of 80%), the sample size of the experimental group and the control group was respectively 197 cases. Considering a dropout rate of 15%, the sample size of each group estimated would be 232 cases (240 cases roughly), therefore we aimed to recruit 480 cases.
