# Supplementary material for: Acupuncture lowering blood pressure for secondary prevention of stroke: a study protocol for a multicenter randomized controlled trial
Source: Trials. 2017 Sep 15;18:428. doi: 10.1186/s13063-017-2171-5 (PMC5603044; doi:10.1186/s13063-017-2171-5)
Supplement: Supplementary file 2 — SPIRIT 2013 Checklist. (DOC 139 kb) [file 13063_2017_2171_MOESM2_ESM.doc]

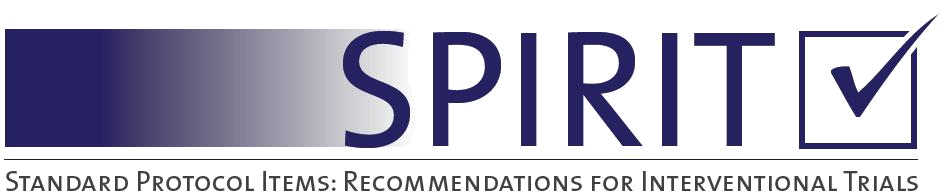


SPIRIT 2013 Checklist: Recommended items to address in a clinical trial protocol and related documents*

| **Section/item** | **Item** | **Description** |
| --- | --- | --- |
|  | **No** |  |
|  | | |
| **Administrative information** | | |
| Title | 1 | Descriptive title identifying the study design, population, interventions, |
|  |  | and, if applicable, trial acronym  **(Dear editors and peer reviewers, please check P1)** |
| Trial registration | 2a | Trial identifier and registry name. If not yet registered, name of |
|  |  | intended registry  **(Please check P3)** |
|  | 2b | All items from the World Health Organization Trial Registration Data |
|  |  | Set |
| Protocol version | 3 | Date and version identifier **(Please check P6)** |
| Funding | 4 | Sources and types of financial, material, and other support **(Please check P21)** |
| Roles and | 5a | Names, affiliations, and roles of protocol contributors **(Please check P1 and P21)** |
| Responsibilities | 5b | Name and contact information for the trial sponsor **(Please check P21)** |
|  |
|  | 5c | Role of study sponsor and funders, if any, in study design; collection, |
|  |  | management, analysis, and interpretation of data; writing of the report; |
|  |  | and the decision to submit the report for publication, including whether |
|  |  | they will have ultimate authority over any of these activities **(Please check P16: Data management and Quality control)** |
|  | 5d | Composition, roles, and responsibilities of the coordinating centre, |
|  |  | steering committee, endpoint adjudication committee, data |
|  |  | management team, and other individuals or groups overseeing the |
|  |  | trial, if applicable (see Item 21a for data monitoring committee)  **(Please check P8 and P15)** |
| **Introduction** |  |  |
| Background and | 6a | Description of research question and justification for undertaking the |
| Rationale |  | trial, including summary of relevant studies (published and |
|  |  | unpublished) examining benefits and harms for each intervention  **(Please check P3-4)** |
|  | 6b | Explanation for choice of comparators**(Please check P5)** |
| Objectives | 7 | Specific objectives or hypotheses **(Please check P5)** |
| Trial design | 8 | Description of trial design including type of trial (eg, parallel group, |
|  |  | crossover, factorial, single group), allocation ratio, and framework (eg, |
|  |  | superiority, equivalence, noninferiority, exploratory) **(Please check P6)** |

**Methods: Participants, interventions, and outcomes**

| Study setting | 9 | Description of study settings (eg, community clinic, academic hospital) |
| --- | --- | --- |
|  |  | and list of countries where data will be collected. Reference to where |
|  |  | list of study sites can be obtained**(Please check P6)** |
| Eligibility criteria | 10 | Inclusion and exclusion criteria for participants. If applicable, eligibility |
|  |  | criteria for study centres and individuals who will perform the |
|  |  | interventions (eg, surgeons, psychotherapists) **(Please check P7-8)** |
| Interventions | 11a | Interventions for each group with sufficient detail to allow replication, |
|  |  | including how and when they will be administered **(Please check P10-11)** |
|  | 11b | Criteria for discontinuing or modifying allocated interventions for a |
|  |  | given trial participant (eg, drug dose change in response to harms, |
|  |  | participant request, or improving/worsening disease) **(Please check P11-12)** |
|  | 11c | Strategies to improve adherence to intervention protocols, and any |
|  |  | procedures for monitoring adherence (eg, drug tablet return, |
|  |  | laboratory tests) **(Please check P13: Outcome assessments)** |
|  | 11d | Relevant concomitant care and interventions that are permitted or |
|  |  | prohibited during the trial **(Please check P11: Control group)** |
| Outcomes | 12 | Primary, secondary, and other outcomes, including the specific |
|  |  | measurement variable (eg, systolic blood pressure), analysis metric |
|  |  | (eg, change from baseline, final value, time to event), method of |
|  |  | aggregation (eg, median, proportion), and time point for each |
|  |  | outcome. Explanation of the clinical relevance of chosen efficacy and |
|  |  | harm outcomes is strongly recommended **(Please check P12-13)** |
| Participant | 13 | Time schedule of enrolment, interventions (including any run-ins and |
| timeline |  | washouts), assessments, and visits for participants. A schematic |
|  |  | diagram is highly recommended (see Figure) **(Please check P27-28)** |
| Sample size | 14 | Estimated number of participants needed to achieve study objectives |
|  |  | and how it was determined, including clinical and statistical |
|  |  | assumptions supporting any sample size calculations**(Please check P14)** |
| Recruitment | 15 | Strategies for achieving adequate participant enrolment to reach |
|  |  | target sample size**(Please check P8-9)** |

**Methods: Assignment of interventions (for controlled trials)**

Allocation:

Sequence 16a Method of generating the allocation sequence (eg, computer-

generation generated random numbers), and list of any factors for stratification.

To reduce predictability of a random sequence, details of any planned

restriction (eg, blocking) should be provided in a separate document

that is unavailable to those who enrol participants or assign

interventions **(Please check P9)**

| Allocation | 16b | Mechanism of implementing the allocation sequence (eg, central |
| --- | --- | --- |
| concealment |  | telephone; sequentially numbered, opaque, sealed envelopes), |
| mechanism |  | describing any steps to conceal the sequence until interventions are |
|  |  | assigned **(Please check P9-10)** |
| Implementation | 16c | Who will generate the allocation sequence, who will enroll participants, |
|  |  | and who will assign participants to interventions **(Please check P9, P7:Participants)** |
| Blinding | 17a | Who will be blinded after assignment to interventions (eg, trial |
| (masking) |  | participants, care providers, outcome assessors, data analysts), and |
|  |  | How **(Please check P10:interventions and P12: Outcome assessments)** |
|  | 17b | If blinded, circumstances under which unblinding is permissible, and |
|  |  | procedure for revealing a participant’s allocated intervention during |
|  |  | the trial **(Please check P15: Patient safety)** |

**Methods: Data collection, management, and analysis**

| Data collection | 18a | Plans for assessment and collection of outcome, baseline, and other |
| --- | --- | --- |
| methods |  | trial data, including any related processes to promote data quality (eg, |
|  |  | duplicate measurements, training of assessors) and a description of |
|  |  | study instruments (eg, questionnaires, laboratory tests) along with |
|  |  | their reliability and validity, if known. Reference to where data |
|  |  | collection forms can be found, if not in the protocol  **(Please check P6: Study design and setting, P16: Data management and Quality control)** |
|  | 18b | Plans to promote participant retention and complete follow-up, |
|  |  | including list of any outcome data to be collected for participants who |
|  |  | discontinue or deviate from intervention protocols**(Please check P13: Outcome assessments)** |
| Data | 19 | Plans for data entry, coding, security, and storage, including any |
| management |  | related processes to promote data quality (eg, double data entry; |
|  |  | range checks for data values). Reference to where details of data |
|  |  | management procedures can be found, if not in the protocol**(Please check P16)** |
| Statistical | 20a | Statistical methods for analysing primary and secondary outcomes. |
| methods |  | Reference to where other details of the statistical analysis plan can be |
|  |  | found, if not in the protocol **(Please check P14-15)** |
|  | 20b |  |
|  |  | Methods for any additional analyses (eg, subgroup and adjusted analyses) **(Please check P15)** |
|  | 20c | Definition of analysis population relating to protocol non-adherence |
|  |  | (eg, as randomised analysis), and any statistical methods to handle |
|  |  | missing data (eg, multiple imputation) **(Please check P14)** |
| **Methods: Monitoring** | |  |
| Data monitoring | 21a | Composition of data monitoring committee (DMC); summary of its role |
|  |  | and reporting structure; statement of whether it is independent from |
|  |  | the sponsor and competing interests; and reference to where further |

details about its charter can be found, if not in the protocol. Alternatively, an explanation of why a DMC is not needed

**(Please check P16)**

21b Description of any interim analyses and stopping guidelines,

including who will have access to these interim results and make the final decision to terminate the trial **(Please check P15:Patient safety)**

Harms 22 Plans for collecting, assessing, reporting, and managing

solicited and spontaneously reported adverse events and

other unintended effects of trial interventions or trial conduct**(Please check P15: Patient safety)**

Auditing 23 Frequency and procedures for auditing trial conduct, if any,

and whether the process will be independent from

investigators and the sponsor**(Please check P16)**

**Ethics and dissemination**

Research ethics

approval 24 Plans for seeking research ethics committee/institutional

review board (REC/旧B) approval **(Please check P8)**

Protocol

Amendments 25 Plans for communicating important protocol modifications (eg,

changes to eligibility criteria, outcomes, analyses) to relevant

parties(eg, investigators, REC/旧Bs, trial participants, trial

registries, journals, regulators) **(Please check P16)**

Consent or assent 26a Who will obtain informed consent or assent from potential trial

participants or authorised surrogates, and how (see Item 32)

**(Please check P9)**

26b Additional consent provisions for collection and use of

participant data and biological specimens in ancillary studies, if

applicable

Confidentiality 27 How personal information about potential and enrolled

participants will be collected, shared, and maintained in order

to protect confidentiality before, during, and after the trial

**(Please check P16: Data management and Quality control)**

Declaration of

Interests 28 Financial and other competing interests for principal

investigators for the overall trial and each study site

**(Please check P21)**

Access to data 29 Statement of who will have access to the final trial dataset, and

disclosure of contractual agreements that limit such access for

investigators **(Please check P16)**

Ancillary and

post-trial care 30 Provisions, if any, for ancillary and post-trial care, and for

compensation to those who suffer harm from trial participation

**(Please check P15-16)**

Dissemination

Policy 31 a Plans for investigators and sponsor to communicate trial results to participants, healthcare professionals, the public, and other relevant groups (eg, via publication, reporting in results databases, or other data sharing arrangements), including any publication restrictions

**(Please check P17)**

31 b Authorship eligibility guidelines and any intended use of

professional writers **(Please check P17)**

31c Plans, if any, for granting public access to the full protocol,

participant-level dataset, and statistical code

Appendices

Informed consent

Material 32 Model consent form and other related documentation given to participants and authorised surrogates

**(Please check P9)**

Biological

Specimens 33 Plans for collection, laboratory evaluation, and storage of biological specimens for genetic or molecular analysis in the current trial and for future use in ancillary studies, if applicable **(Please check P17)**

|  |
| --- |

*It is strongly recommended that this checklist be read in conjunction with the SPIRIT 2013 Explanation&Elaboration for important clarification on the items. Amendments to the protocol should be tracked and dated. The SPIRIT checklist is copyrighted by the SPIRIT Group under the Creative Commons "Attribution一Noncommercial一NoDerivs 3.0 Unported" license.
